# Cost-effectiveness of procalcitonin for detection of serious bacterial infections in children presenting with fever without source

**DOI:** 10.1186/s12887-022-03293-3

**Published:** 2022-04-26

**Authors:** Jefferson Antonio Buendía, Diana Guerrero Patiño

**Affiliations:** 1 Research Group in Pharmacology and Toxicology ”INFARTO”, Department of Pharmacology and Toxicology, University of Antioquia, Medellín, Colombia; 2 Hospital Infantil Concejo de Medellín, Medellín, Colombia

**Keywords:** Bacterial Infections, Child, Colombia

## Abstract

**Introduction:**

Procalcitonin (PCT) offers better specificity than C-reactive protein (CRP) to detect SBI. However, their cost limited their use and routine application. The objective of this work is to determine the cost-effectiveness of PCT against CPR or Rochester scale in infants between 1 and 3 months from the perspective of the third payer in Colombia.

**Methods:**

A Monte Carlo simulation was performed with a hypothetical cohort of 10,000 patients with fever without focus (FWS) between 1 to 3 months, to estimate the number of cases correctly diagnosed for each test and the associated costs with each test.

**Results:**

The test with the highest number of correctly diagnosed cases was PCT 79%, followed by C-reactive protein 75%, and the Rochester scale 68%. The test with the lowest cost per patient was PCT $645 (95% CI US$646-US$645) followed by C-reactive protein U$ 653 (95% CI US$655-$645) and Rochester scale US$804 (95% CI US$807-US$804). This position of dominance of PCT eliminated the need to calculate an incremental cost effectiveness ratio.

**Conclusions:**

PCT is the most cost-effective strategy for the detection of IBS in infants with FWS. These results should be interpreted within the clinical context of the patient and not as a single method for therapeutic decision-making.

## Introduction

Fever is one of the most frequent signs of illness in pediatric practice [1, 2]. In infants under 3 months of age, fever could be the only manifestation of disease without any finding on physical examination [2]. The infants with this fever without focus (FWS) have a good prognosis, and most of the cases are self-limited [3]. However, between 1 to 30% of the patients will develop some severe bacterial infection (SBI) such as urinary tract infection, bacteremia, pneumonia, or meningitis [3]. One of the current challenges in FWS is to reduce clinical heterogeneity in diagnosis. There is a recognized gap between the evidence on diagnostic tests and their use in real life. It is still common to use invasive diagnostic tests indiscriminately regardless of individual risk of SBI [4, 5]. Different test has historically been proposed for the detection of IBS such as procalcitonin (PCT) and C-reactive protein (CRP). PCT offers better specificity than CRP to detect SBI with an early detection possible of SBI if the evolution of the fever is < 12 h [6]. However, their cost limited their use in developing countries [7, 8]. Economic evaluations are tools that provide objective information to decision-makers regarding the inclusion of new diagnostic tests in health insurance plans. Demonstrating or not the cost-effectiveness of PCT with respect to routine tests such as CPR or clinical scales such as the Rochester scale would allow deciding whether the clinical benefit provided by PCT is outweighed by its actual cost. The objective of this work is to determine the cost-effectiveness of PCT against routine management based on the CPR or Rochester scale in infants between 1 and 3 months from the perspective of the third payer in Colombia.

## Methodology

### Patients and methods

**Fig. 1:**
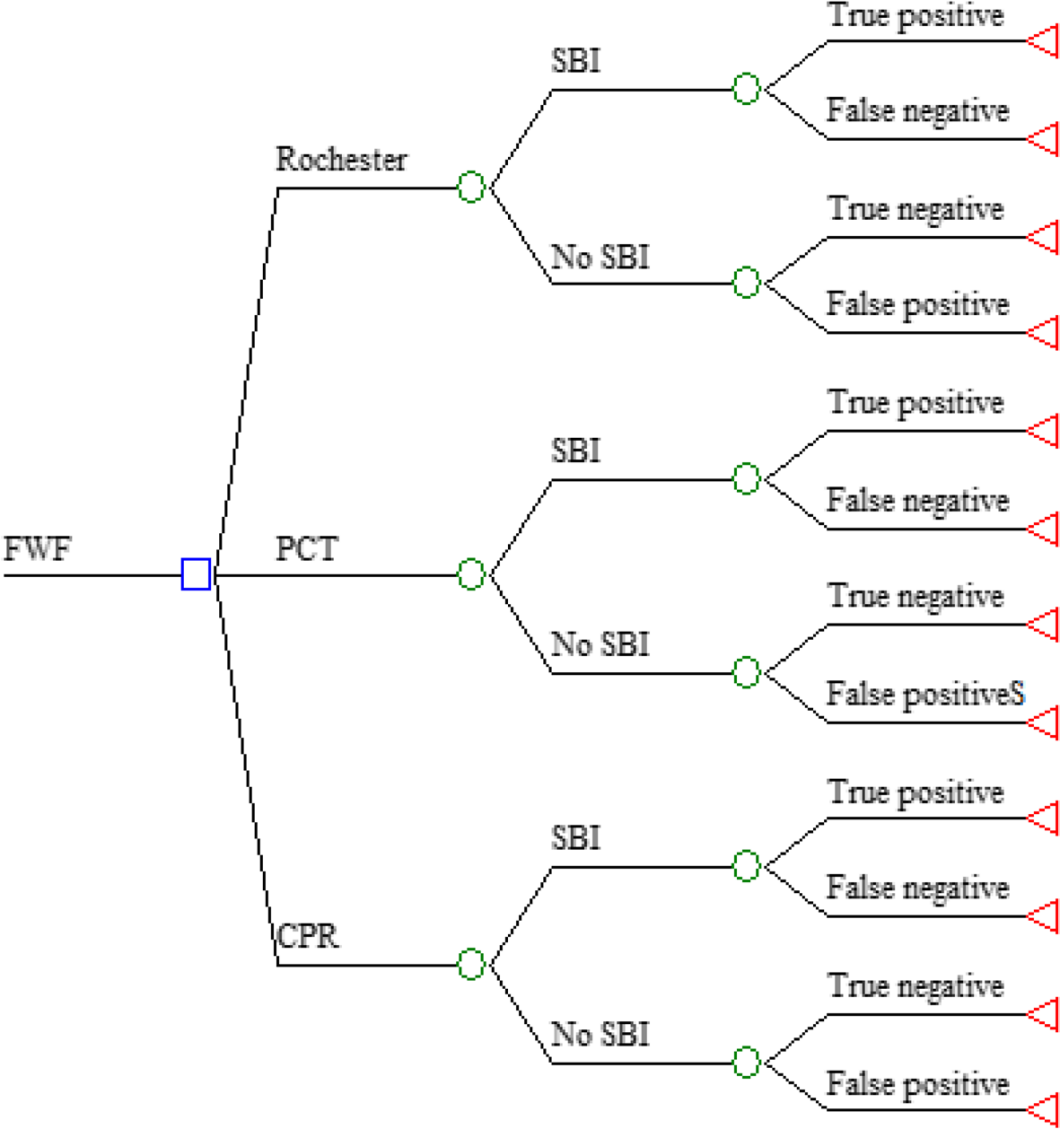
Markov model

An economic model of cost-effectiveness using the decision analysis technique (decision tree) was designed to select the best strategy for identifying SBI ( defined as sepsis, bacterial meningitis, pneumonia or urinary tract infection) in infants between 1 to 3 months with FWS (defined as the presence of fever in a patient in the age range mentioned in whom the etiology could not be established after a medical history and detailed physical examination) [3, 9]. The tests evaluated were C-reactive protein, procalcitonin and application of the Rochester predictive scale which is the reference test in current local clinical practice [10], Fig. 1. The sensitivity and specificity data of each of the tests were extracted from systematic reviews with meta-analysis for each of the respective tests to be evaluated, see Table 1. We considered as positive IBS values: the presence of a procalcitonin value equal or greater than 2 ng/mL [11], a CPR value equal or greater than 40 mg/l [12], or patient classified by Rochester scale as high-risk criteria (that is, the non-fulfillment of any of the following conditions: previously healthy child without perinatal complications and without previous antibiotic therapy, normal physical examination, white blood cell count between 5000–10,000 per mm3, urinalysis with the presence of less than 10 white blood cells per field in centrifuged urine) [13]. The clinical data related to the frequency of IBS used in this study were based Colombian studies of prevalence of SBI in infants [10].

**Table 1:** Base case of study

Variable	Base case	Valor Low	Valor High	Reference
Probability of SBI	0,41	0,308	0,513	10
Rochester scale Sensitivity	0,96	0,717	1,000	13
Rochester scale Specificity	0,49	0,368	0,613	13
CPR Sensitivity	0,74	0,555	0,925	12
CPR Specificity	0,76	0,570	0,950	12
PCT Sensitivity	0,61	0,458	0,763	11
PCT Specificity	0,94	0,705	1,000	11
Costs of a true positive SBI case	1050	788	1313	
Costs of a true false negative case of SBI	1946	1122	1870	
Costs of a true false positive case of SBI	883	625	1041	
Cost of PCT	68	51	85	
Cost of Rochester scale	17	13	21	
Cost of CPR	10	8	13	

### Costs included

The perspective of the study was of the third-party payer incorporating only the direct costs of treatment, hospitalization, and diagnosis. The direct micro costing technique was used to determine the hospitalization expenditure per day of hospitalization, medications, diagnostic tests (blood count, urine cultures, blood cultures, uranalysis, CSF culture, PCR, PCL), diagnostic images (chest x-ray, renal ultrasound), medical and nursing procedures. Medical direct costs were valued at market prices, using as reference standard fees from Colombia´s Social Security manual. Generally, contracts between insurers and providers of health services is based on this national tariff manual [14]. All information were evaluated by a group of experts (head of the pediatrics service, pediatric infectologist, head of purchases and supplies, and medical auditors of the service); and which also agreed on the range of lower and higher values for each cost. All costs were expressed in US dollars (Exchange rate 01/06/21, 1 US$ = COL $ 3800) [14]. The cost of the Rochester scale included the cost of a blood count and a complete urine test with urine culture. The cost of procalcitonin and C-reactive protein included the direct costs of testing. The cost of hospitalization of false positive for SBI included the costs of medicines, diagnostic tests, and other costs described above during 3 days of hospitalization, period after which blood culture and urine culture readings are obtained with which hospital discharge is granted in patients with good clinical evolution and negative results in said cultures. The cost of hospitalization of a false negative included the costs of a first hospitalization for 3 days, such as those mentioned above for the case of a false positive, and the costs of a second hospitalization for the management of an SBI (cost of false positive + cost of true positive)  [8]; only including management costs in pediatric hospitalization without including derived from care in pediatric intensive care unit given the low rate of complications and mortality associated with SBI in infants with FSFS reported both locally and internationally [9, 10]. Indirect costs (e.g., absenteeism costs, etc.) and direct non-medical costs (transportation, etc.) were not considered. Cost-effectiveness was evaluated at a willingness-to-pay (WTP) value of US$5180 [15].

### Sensitivity analysis

We conduct a one-way sensitivity presenting these results in the tornado diagram. Probabilistic sensitivity analysis was also performed. For this purpose, random sampling was performed from each of the parameter distributions. We used the beta distribution for probabilities and the gamma distribution for costs. For each treatment strategy, we calculated the expected costs and highest number of correctly diagnosed cases (true positives + true negatives) using the combination of all parameter values in the model. To do this calculation, a second-order Monte Carlo simulation with 10,000 replications of each parameter was made: resulting in the expected cost-effectiveness for each treatment strategy. To represent decision uncertainty, we plot the cost-effectiveness and acceptability frontiers. Microsoft Excel® was used in all analyses.

## Results

### Number of correctly diagnosed cases and average costs per patient in each strategy

Based on the Monte Carlo simulation, the test with the highest number of correctly diagnosed cases (true positives + true negatives) was PCT (7942/10000 correctly diagnosed cases, 95% confidence interval: 7944—7941), followed by C-reactive protein (7577/10000 correctly diagnosed cases, 95% confidence interval: 7578—7576), and the Rochester scale (6768/10000 correctly diagnosed cases, 95% confidence interval: 6769—6767). Regarding the test with the lowest cost per patient was PCT $645 (95% CI US$646-US$645) followed by C-reactive protein U$ 653 (95% CI US$655-$645) and Rochester scale US$804 (95% CI US$807-US$804), Table 2.

**Table 2:** Cost effectiveness of PCT, CPR and Rochester scale

Estrategia	Cost (**US$)**	Difference	Correctly diagnosed case of SBI	Difference	Cost / effectiveness **(US$)**	Category
PCT	645	-159	79%	12%	812	
CPR	653	-151	76%	8%	862	Absolutely dominated
Rochester	804	Reference	68%	Reference	1188	Absolutely dominated

*PCT* Procalcitonin, *CRP* C-reactive protein

### Cost analysis – incremental effectiveness

When performing the cost-effectiveness analysis, it was observed that the use of PCT was the most cost-effective strategy by obtaining a negative cost-effectiveness ratio of per correctly diagnosed case compared to Rochester scale and PCR, see Table 2. A position of dominance eliminated the need to calculate an incremental cost effectiveness ratio. In both cases PCT obtained a higher number of correctly diagnosed cases with a lower cost per patient.

### Sensitivity analysis

**Fig. 2:**
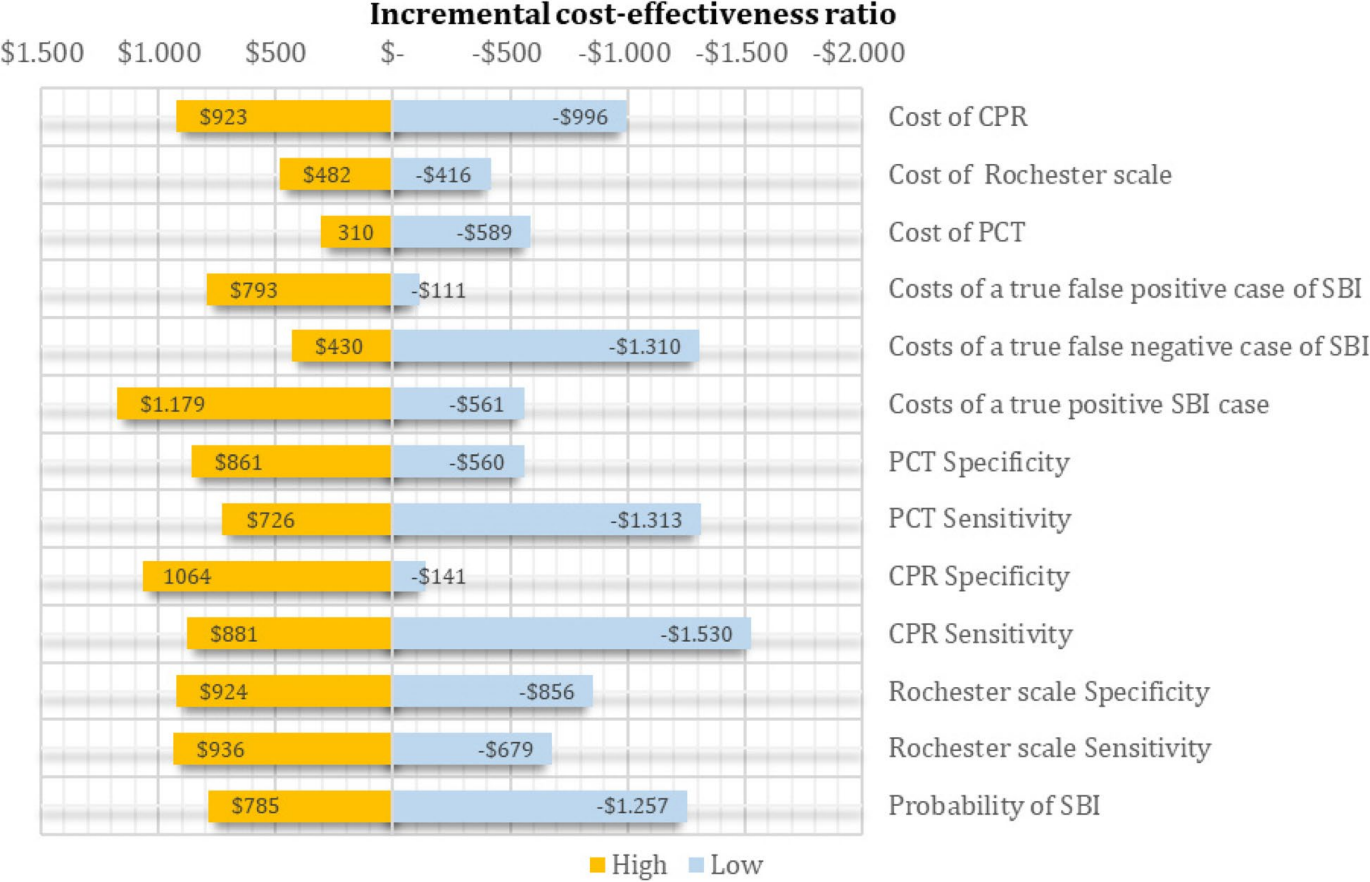
Tornado diagram

**Fig. 3:**
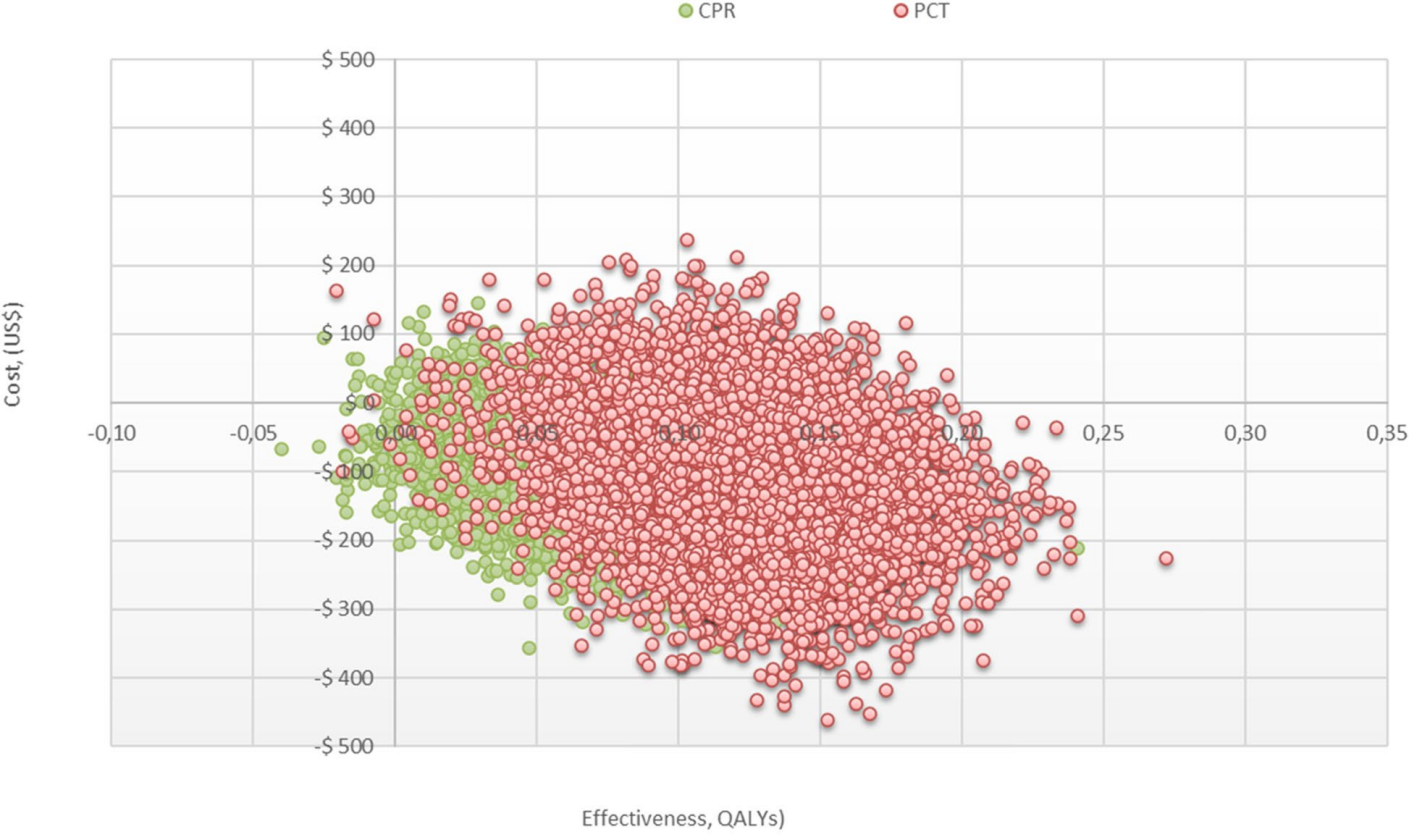
Cost-effectiveness plane

In the deterministic sensitivity analyses, our base case results were robust to variations in all assumptions and parameters. That is, changing each of the parameters, within the ranges mentioned in the methods section, of cost and transition probabilities did not alter the incremental cost-effectiveness ratio (between PCT and Rochester scale) significantly or change its interpretation. For none of the variables evaluated, variations within the established ranges led to the incremental cost-effectiveness ratio being higher than the willingness to pay (WTP) in Colombia, Fig. 2. The results of probabilistic sensitivity analysis are graphically represented on the cost-effectiveness plane in Fig. 3. This scatter plot shows that PCT and CPR, compared with to Rochester, tends to be associated with lower costs and higher number of correctly diagnosed cases. Indeed, 88% of simulations of PCR were graphed in quadrant 2 (lower cost, high QALYs), 12% in quadrant 1 (high cost, high QALYs).

## Discussion

Fever continues to be a frequent cause of medical attention in the population given the anxiety it generates in parents. Medical errors related to a timely diagnosis of SBI continue to occur in pediatric practice. It is precisely in this scenario where it becomes important to have tools that allow detecting the largest number of SBI cases with the lowest possible cost given the context of limited resources in our health system [16–19].

In our study we found that PCT was the most cost-effective strategy for the detection of IBS with respect to CPR and the application of the Rochester scale. This test was associated in our study with lower cost per correctly diagnosed patient respect to CPR and Rochester scale. While the difference in the proportion of correctly diagnosed cases of PCT versus the Rochester scale was 14%, versus CPR was 4%. Likewise, while the difference in cost per correctly diagnosed case for PCT versus the Rochester scale was $159, versus CPR was $9. This short difference between PCT and CPR was also evident in the Monte Carlo simulation where the point cloud in the cost-effectiveness plane between these tests is overlapping.

None of the tests evaluated reached proportions of correctly diagnosed patients greater than 90%; which undoubtedly limits their use as unique tests for therapeutic decision-making; and should always be interpreted in the clinical context of the patient as expressed in the clinical practice guidelines of this entity [4, 20–22]. In the deterministic sensitivity analyses, our base case results were robust to variations in all assumptions and parameters. Within the different ranges evaluated of sensitivity and specificity that each test, there was no change with respect to the cost effectiveness of PCT respect to other tests.

Rochester scale had a lower the proportion of correctly diagnosed cases than C-reactive protein and CRP; which is due to the large number of false positives that increase [20, 23]. The CPR has a later increase (± 4–6 h.) compared to PCT, and this can explain the small differences in the number of cases correctly diagnosed. However, to evaluate the impact that early consultation (< 4 h) would have on the effectiveness and cost-effectiveness of the tests evaluated is beyond the scope of this work. The panorama in the comparative studies, where most patients come after 4–6 h of the start of symptoms, is not different from the real life providing greater external validity to our results [24]. We previously published an economic evaluation of these same three tests in Argentina in 2013 [7]. This study found small differences between CPR and PCT with respect to their cost-effectiveness. In our study the values of sensitivity and specificity of PCT (0.61 and 0.94 respectively) were extracted from a meta-analysis of 12 studies while the Argentine study extracted it from a meta-analysis of 6 studies (0.71 and 0.80 respectively). It is precisely this gain in specificity, and the consequent reduction of false positives, which may explain the differences between these studies, in addition to a different cost structure in both countries.

## Limitations

The present work has several limitations. We use data extracted from the literature and not estimated directly from our population. As was mentioned previously, the reliability and robustness of the results were evaluated by sensitivity analysis. The changing each of these parameters, within their ranges did not alter the incremental cost-effectiveness ratio significantly or change its interpretation. The direct micro costing technique was used to determine the direct cost and cannot exclude selection or information bias in these values. However, the incremental cost-effectiveness ratio estimate was robust to any variation in the cost in the study. The objective of our study is to evaluate the individual cost effectiveness of these tests and not to evaluate different diagnostic algorithms. Our study provides evidence for future evaluation of diagnostic algorithms where combinations of these or other tests are tested.

In conclusion, PCT is the most cost-effective strategy in Colombia for the detection of IBS in infants who come with SWS However, since these tests as well as the others evaluated do not have high proportions of correctly diagnosed cases; they should be interpreted within the clinical context of the patient and not as a single method for therapeutic decision-making.

## Data Availability

Database fever without source [Data set]. Zenodo. https://doi.org/10.5281/zenodo.5841237

## References

[1] Phasuk N, Nurak A (2020). Etiology, treatment, and outcome of children aged 3 to 36 months with fever without a source at a community hospital in Southern Thailand. J Prim Care Community Health.

[2] Davis T (2013). NICE guideline: feverish illness in children–assessment and initial management in children younger than 5 years. Arch Dis Child Educ Pract Ed.

[3] Baraff LJ (2000). Management of fever without source in infants and children. Ann Emerg Med.

[4] Van den Bruel A, Thompson MJ, Haj-Hassan T, Stevens R, Moll H, Lakhanpaul M (2011). Diagnostic value of laboratory tests in identifying serious infections in febrile children: systematic review. BMJ.

[5] Van den Bruel A, Thompson M, Buntinx F, Mant D (2012). Clinicians' gut feeling about serious infections in children: observational study. BMJ.

[6] Hu L, Shi Q, Shi M, Liu R, Wang C (2017). Diagnostic value of PCT and CRP for detecting serious bacterial infections in patients with fever of unknown origin: a systematic review and meta-analysis. Appl Immunohistochem Mol Morphol.

[7] Antonio Buendía J, Colantonio L (2013). Costo-Efectividad de la Proteína C Reactiva, Procalcitonina y Escala de Rochester: Tres Estrategias Diagnosticas para la Identificación de Infección Bacteriana Severa en Lactantes Febriles sin Foco. Value in Health Reg Issues.

[8] Buendia JA, Sanchez-Villamil JP, Urman G (2016). Cost-effectiveness of diagnostic strategies of severe bacterial infection in infants with fever without a source. Biomedica.

[9] Baraff LJ (2008). Management of infants and young children with fever without source. Pediatr Ann.

[10] Gutierrez IFOJ, Rodriguez T, Zamora F (2012). Fiebre sin foco aparente en menores de 36 meses en un servicio de urgencias de un hospital de tercer nivel de Bogotá. Colombia Pediatria.

[11] Trippella G, Galli L, De Martino M, Lisi C, Chiappini E (2017). Procalcitonin performance in detecting serious and invasive bacterial infections in children with fever without apparent source: a systematic review and meta-analysis. Expert Rev Anti Infect Ther.

[12] Yo CH, Hsieh PS, Lee SH, Wu JY, Chang SS, Tasi KC (2012). Comparison of the Test Characteristics of Procalcitonin to C-Reactive Protein and Leukocytosis for the Detection of Serious Bacterial Infections in Children Presenting With Fever Without Source: A Systematic Review and Meta-analysis. Ann Emerg Med.

[13] Jaskiewicz JA, McCarthy CA, Richardson AC, White KC, Fisher DJ, Dagan R (1994). Febrile infants at low risk for serious bacterial infection–an appraisal of the Rochester criteria and implications for management Febrile Infant Collaborative Study Group. Pediatrics.

[14] Ministerio de Salud. Decreto 2423, manual tarifario SOAT 2021. Available from: https://consultorsalud.com/wp-content/uploads/2020/12/Manual-Tarifario-SOAT-de-Salud-2021-Consultorsalud.pdf. Accessed 24 Apr 2022.

[15] Espinosa O, Rodríguez-Lesmes P, Orozco L, Ávila D, Enríquez H, Romano G, Ceballos M. Estimating cost-effectiveness thresholds under a managed healthcare system: experiences from Colombia. Health Policy Plan. 2022;37(3):359–68. 10.1093/heapol/czab146. 10.1093/heapol/czab146 34875689

[16] Van den Bruel A, Bartholomeeusen S, Aertgeerts B, Truyers C, Buntinx F (2006). Serious infections in children: an incidence study in family practice. BMC Fam Pract.

[17] Klein-Kremer A, Goldman RD (2011). Return visits to the emergency department among febrile children 3 to 36 months of age. Pediatr Emerg Care.

[18] Najaf-Zadeh A, Dubos F, Aurel M, Martinot A (2008). Epidemiology of malpractice lawsuits in paediatrics. Acta Paediatr.

[19] Iglesias CP, Drummond MF, Rovira J (2005). Health-care decision-making processes in Latin America: problems and prospects for the use of economic evaluation. Int J Technol Assess Health Care.

[20] Thompson M, Van den Bruel A, Verbakel J, Lakhanpaul M, Haj-Hassan T, Stevens R (2012). Systematic review and validation of prediction rules for identifying children with serious infections in emergency departments and urgent-access primary care. Health Technol Assess.

[21] NICE. Feverish illness in children: National Institute for Health and Clinical Excellence. Available from: http://guidance.nice.org.uk/CG160. Accessed 24 Apr 2022.

[22] Montero D, Miron L, Cheistewer A. Medicina interna para pediatras. Guia practica. Hospital de Niños Ricardo Gutierrez. Buenos Aires: Pfzier; 2010. Available from: http://www.afam.org.ar/textos/03_10_18/guia_medicina_interna_pediatra_2018.pdf. Accessed 24 Apr 2022.

[23] Huppler AR, Eickhoff JC, Wald ER (2010). Performance of low-risk criteria in the evaluation of young infants with fever: review of the literature. Pediatrics.

[24] Elshout G, Monteny M, van der Wouden JC, Koes BW, Berger MY (2011). Duration of fever and serious bacterial infections in children: a systematic review. BMC Fam Pract.

